# Design of a phase II randomised, double-blind, placebo-controlled, dose-finding trial of BI 1819479 in patients with idiopathic pulmonary fibrosis

**DOI:** 10.1183/23120541.00973-2025

**Published:** 2026-03-16

**Authors:** Wim A. Wuyts, Francesco Bonella, Haruyuki Ishii, Joyce S. Lee, Elisabetta Renzoni, Sandra Hadl, Julia Krzykalla, Susanne Stowasser, Michael Engel, Maria Molina Molina

**Affiliations:** 1 Unit for Interstitial Lung Diseases, Department of Respiratory Diseases, University Hospitals Leuven, Leuven, Belgium; 2 Department of Chronic Diseases, Metabolism, and Ageing, KU Leuven, Leuven, Belgium; 3 Center for Interstitial and Rare Lung Diseases, Pneumology Department, Ruhrlandklinik, University Hospital, University of Essen, European Reference Network (ERN)-LUNG, ILD Core Network, Essen, Germany; 4 Department of Respiratory Medicine, Kyorin University Faculty of Medicine, Mitaka City, Japan; 5 University of Colorado, School of Medicine, Department of Medicine, Aurora, CO, USA; 6 Interstitial Lung Disease Unit, Royal Brompton and Harefield Clinical Group, Guy's and St Thomas’ NHS Foundation Trust, London, UK; 7 Margaret Turner Warwick Centre for Fibrosing Lung Disease, National Heart and Lung Institute, Imperial College London, London, UK; 8 Boehringer Ingelheim RCV & Co. KG, Vienna, Austria; 9 Boehringer Ingelheim Pharma & Co. KG, Biberach an der Riss, Germany; 10 Boehringer Ingelheim Corporation, Ingelheim am Rhein, Germany; 11 Interstitial Lung Disease Unit, Respiratory Department, University Hospital of Bellvitge, IDIBELL, CIBERES, Barcelona, Spain

## Abstract

**Background:**

Current treatments for idiopathic pulmonary fibrosis (IPF) slow but do not stop/reverse disease progression. The lysophosphatidic acid (LPA) axis is identified as a therapeutic target for IPF.

**Objective:**

This study aims to assess BI 1819479, an LPA pathway inhibitor, in patients with IPF (ClinicalTrials.gov Identifier: NCT06335303).

**Methods:**

In this placebo-controlled, phase II trial, patients will be randomised (2:1:1:1) to receive one of three oral doses of BI 1819479 or placebo, stratified by nintedanib/pirfenidone use. Patients aged ≥40 years with IPF, forced vital capacity (FVC) ≥45% of predicted normal and haemoglobin-corrected diffusing capacity for carbon monoxide ≥25% of predicted normal at screening will be included. Patients with relevant airway obstruction (pre-bronchodilator forced expiratory volume in 1 s/FVC <0.7), acute IPF exacerbation ≤12 weeks prior to screening, treatment with immunosuppressive medications (other than oral corticosteroids) or prednisone >15 mg·day^−1^ or equivalent, will be excluded. Treatment with approved IPF treatments (nintedanib/pirfenidone) is allowed if at a stable dose for ≥12 weeks prior to trial entry. Patients will be treated until completing 52 weeks, or 24 weeks after the last patient is randomised, whichever occurs first. The primary end-point is the annual rate of FVC decline (mL·year^−1^) assessed up to 52 weeks; the secondary end-point is absolute change from baseline in FVC at week 24. Safety will be assessed throughout.

**Conclusion:**

This trial evaluates the efficacy, safety and dose range of BI 1819479 in patients with IPF, offering a potential additional treatment option, and will establish appropriate dosing for phase III trials.

## Shareable abstract

**This trial will evaluate the efficacy, safety and dose range of a lysophosphatidic acid pathway inhibitor in patients with IPF, offering potential additional treatment options for patients** https://bit.ly/4qqnRJx

## Background

Idiopathic pulmonary fibrosis (IPF) is a progressive fibrosing interstitial lung disease (ILD) with a variable and unpredictable course [1, 2]. The disease trajectory is characterised by increasing respiratory symptoms, worsening pulmonary function, unpredictable episodes of acute respiratory decline, and ultimately death [2].

Nintedanib and pirfenidone are currently the only approved treatments for IPF and are the recommended standard of care per American Thoracic Society (ATS)/European Respiratory Society (ERS)/Japanese Respiratory Society (JRS)/Latin American Thoracic Association (ALAT) 2022 clinical practice guidelines [3–5]. However, while these treatments can slow disease progression, they do not stop/reverse pulmonary fibrosis [6]. Individual clinical trials with these approved treatments have not been powered to show mortality benefit, but analyses of pooled data from clinical trials and observational studies suggest improvement in life expectancy [6]. However, real-world data from heterogeneous IPF registries have shown that only 55–62% of patients diagnosed with IPF were receiving treatment with nintedanib or pirfenidone at enrolment [7]. In untreated patients, the median survival time after diagnosis is approximately 3–5 years [8–10]. Therefore, there remains a high unmet need for more efficacious and better-tolerated treatments in combination with standard of care or as a monotherapy for patients living with IPF.

Elevated levels of lysophosphatidic acid (LPA) and autotaxin have been observed in patients with IPF [11, 12]. LPA is a bioactive lipid involved in many physiological and pathological processes that activate LPA receptors, which have been implicated in the development of IPF [12–14]. LPA drives profibrotic and inflammatory processes by inducing cellular responses, including proliferation, migration, inflammation, and ultimately fibrosis [10, 12]. Studies have demonstrated LPA-induced cellular responses in fibroblasts that enhance the survival and activation of macrophages, which contribute to the fibrotic process [12, 15–17]. Fibroblast activation increases autotaxin secretion, an extracellular enzyme that catalyses the biosynthesis of LPA, creating a positive feedback loop that sustains and amplifies the fibrotic response [10, 15]. Therefore, LPA and autotaxin have been identified as therapeutic targets for the treatment of IPF.

Despite the failure of large phase III clinical trials with ziritaxestat and a phase II clinical trial with BBT-877 in patients with IPF, other compounds targeting the autotaxin–LPA pathway are currently being investigated in phase II and III clinical trials [18–24]. BI 1819479 is a very potent small molecule that inhibits the LPA pathway. Oral treatment with BI 1819479 was shown to have acceptable safety and tolerability in preclinical studies, and therefore advanced to four phase I studies with healthy volunteers [25–28]. Here, we describe the trial design of the phase II trial of BI 1819479 in patients with IPF.

## Materials and methods

### Trial design

**FIGURE 1:**
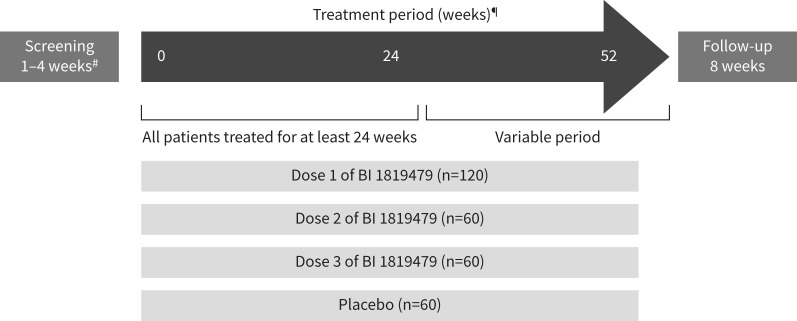
Trial design. ^#^: Screening may be extended up to 6 weeks to account for possible missing eligibility results, administrative, or organisational reasons. ^¶^: Treatment remains the same during the whole treatment period; trial patients will end treatment at different time points, starting from week 24 up to week 52.

This is a phase II (ClinicalTrials.gov Identifier: NCT06335303), randomised, double-blind, placebo-controlled, dose-finding trial evaluating the efficacy, safety and tolerability of three different oral doses of BI 1819479 in patients with IPF (figure 1). This multicentre trial will include approximately 300 enrolled patients across 28 countries (supplementary figure S1). Screening will be conducted to determine eligibility (visit 1). Patients will be randomised at the start of the study period (visit 2), which will allow for the collection of clinical and safety information and review of all inclusion and exclusion criteria. Trial participants will be randomised in a 2:1:1:1 ratio to one of four treatment groups: dose one of BI 1819479, dose two of BI 1819479, dose three of BI 1819479, or placebo, respectively. All trial participants will receive treatment and undergo assessments for a minimum of 24 weeks (visit 2 to visit 8). Following this period, patients will continue the same treatment and have assessments up to 52 weeks. Patients will be treated until either they complete 52 weeks of treatment or until 24 weeks after randomisation of the last patient (end-of-treatment visit within 2 weeks), whichever comes first. After their end-of-treatment visit, patients will receive a follow-up phone call after 4 weeks (±7 days) and have a follow-up (end-of-trial) visit after a minimum of 8 weeks (±3 days). Extending treatment beyond 24 weeks allows for the collection of more long-term efficacy data (more stable treatment estimate of the primary end-point) and long-term safety data without prolonging the trial.

Randomisation will be stratified by background use of currently approved IPF treatments (with or without nintedanib/pirfenidone). The subgroup without background treatment will include patients not receiving nintedanib/pirfenidone in the 12 weeks prior to trial entry. Patients who are on a stable dose of nintedanib/pirfenidone for at least 12 weeks prior to trial entry and plan to remain on this treatment for the duration of the trial will constitute the subgroup with background IPF treatment. Randomisation will be monitored to achieve a distribution of patients of at least 20% without background treatment with nintedanib/pirfenidone and approximately 80% with background nintedanib/pirfenidone.

Subgroup analyses will include sex, age and the background treatment at baseline. In addition, the subgroup of patients receiving background treatment will be further partitioned into patients receiving each specific treatment (*i.e.* nintedanib/pirfenidone).

A summary of the trial design is provided as an infographic in supplement S1.

### Discontinuation of treatment

Patients may discontinue trial treatment temporarily or permanently. Those who permanently discontinue will perform an end-of-treatment visit at the time of discontinuation, followed by a safety follow-up phone call 4 weeks (±7 days) later and an end-of-trial visit at minimum 8 weeks (±3 days) after the last dose. Temporary interruption of BI 1819479 is permitted for specific cases (*e.g.* surgery, adverse events or other diseases), use of restricted concomitant medications or hepatic injury. Resumption of treatment is permitted if medically justified. Additional information is provided in supplement S2.

### Patient population

All inclusion and exclusion criteria are reported in table 1. Patients aged ≥40 years will be eligible for the trial if they have an IPF diagnosis based on ATS/ERS/JRS/ALAT 2022 guidelines, as confirmed by the investigator by chest high-resolution computed tomography (HRCT) scan with usual interstitial pneumonia (UIP) or probable UIP pattern within a clinical context consistent with a diagnosis of IPF [4]. Before randomisation, all HRCT scans will be sent for central review. Patients are also required to have forced vital capacity (FVC) ≥45% of predicted normal at screening and diffusing capacity of the lungs for carbon monoxide (*D*_L__CO_) corrected for haemoglobin ≥25% of predicted normal at screening. Patients with and without background treatment (nintedanib/pirfenidone) will be included. Patients who are on a stable background treatment with nintedanib/pirfenidone at the time of screening and randomisation are planned to continue this background treatment during the trial. Trial patients not receiving approved background treatment for IPF at enrolment and randomisation will be allowed to initiate an approved treatment in case of IPF progression and/or exacerbation (by investigator judgement) after the first 12 weeks of treatment.

**TABLE 1:** Inclusion and exclusion criteria

Inclusion criteria
Patients ≥40 years old at the time of signed informed consent. Signed and dated written informed consent in accordance with ICH GCP and local legislation prior to admission to the trial. Diagnosis of IPF: o based on the 2022 ATS/ERS/JRS/ALAT Guideline as confirmed by the investigator based on chest HRCT scan taken within 12 months of screening and, if available, surgical lung biopsy or transbronchial lung cryobiopsy, o and “UIP” or “Probable UIP” HRCT pattern consistent with the clinical diagnosis of IPF, as confirmed by central review prior to randomisation. ▪ Patients with “Indeterminate for UIP” HRCT findings are eligible if a clinical diagnosis of IPF can be confirmed locally based on (historical) surgical lung biopsy or transbronchial lung cryobiopsy demonstrating a “UIP” or “Probable UIP” histopathology pattern. ▪ Trial patients with HRCT findings suggestive of an “alternative diagnosis” are eligible if a clinical diagnosis of IPF can be confirmed locally based on (historical) surgical lung biopsy or cryobiopsy demonstrating a “UIP” histopathology pattern. Patients may be either: o on stable therapy^#^ with nintedanib or pirfenidone for at least 12 weeks prior to screening and planning to stay on this background treatment after randomisation. Combination of nintedanib plus pirfenidone is not allowed; o not on treatment with nintedanib or pirfenidone for at least 12 weeks prior to screening period (*e.g.* either AF-treatment naive or previously discontinued) and not planning to start or re-start background AF treatment. FVC≥45% of predicted normal at screening. *D*_L__CO_≥25% of predicted normal corrected for haemoglobin at screening. Women of childbearing potential (WOCBP)^¶^ must use highly effective methods of birth control with low user dependency and additional barrier contraception for male partners (use of condom) until end of follow-up period. The WOCBP must conduct regular pregnancy testing (at least monthly) and the trial medication must be discontinued in case of pregnancy. Male trial patients with WOCBP partners must use contraception (condom) to avoid exposure *via* seminal fluid. Female partners of male trial patients must use highly effective methods of contraception during treatment until end of follow-up period.

Exclusion criteria
Acute exacerbation of IPF within at least 12 weeks prior to screening and/or during the screening period (investigator-determined). Treated with immunosuppressive medications (other than oral corticosteroids) or prednisone >15 mg·day^−1^ or equivalent for respiratory or pulmonary reasons. Patients who must or wish to continue the intake of restricted medications (table 2) or any drug considered likely to interfere with the safe conduct of the trial. The patient is currently enrolled in another investigational device or drug trial, or their screening occurs less than 30 days or five half-lives (whichever is longer) after completing a previous investigational device or drug trial or receiving other investigational treatments. Patients with a significant disease or condition other than the IPF under trial, which, in the opinion of the investigator, may put the patient at risk because of participation, interfere with trial procedures or cause concern regarding the patient's ability to participate in the trial or any medical condition that could lead to a life expectancy <12 months. Relevant airways obstruction (pre-bronchodilator FEV_1_/FVC <0.7) at screening. In the opinion of the investigator, other clinically significant pulmonary abnormalities. Lower respiratory tract infection requiring treatment within 4 weeks prior to screening. Any documented active or suspected malignancy or history of malignancy within 5 years prior to screening, except appropriately treated basal cell carcinoma or squamous cell carcinoma of the skin, or *in situ* carcinoma of uterine cervix. Major surgery (major according to the investigator's assessment) performed within 3 months prior to screening or planned during the course of the trial. Being on a lung transplant list is allowed. Patients not expected to comply with the protocol requirements or not expected to complete the trial as scheduled (*e.g.* chronic alcohol or drug abuse or any other condition that, in the investigator's opinion, makes the patient an unreliable trial patient). Previous randomisation in this trial; re-screening is allowed. Hypersensitivity to the drug or its excipients. Women who are pregnant, breastfeeding, or plan to become pregnant while in the trial. Any vulnerable person (defined as: pregnant or breastfeeding women; persons deprived of their liberty; minors; persons who may have insufficient power, intelligence, education, resources, strength, or other needed attributes to protect their own interests; or unable to explicitly give consent), as per local regulation. Other clinically significant concomitant disease state (*e.g.* renal failure, hepatic dysfunction or cardiovascular disease) in the opinion of the investigator. Any condition not covered by any of the other exclusion criteria which, in the investigator's opinion, might jeopardise the patient's safety or compliance with the protocol. History of stem cell therapy for the treatment of pulmonary fibrosis.

AF: antifibrotic; ALAT: Latin American Thoracic Association; ATS: American Thoracic Society; *D*L_CO_: diffusing capacity of the lungs for carbon monoxide; ERS: European Respiratory Society; FEV_1_: forced expiratory volume in 1 s; FVC: forced vital capacity; GCP: Good Clinical Practice; HRCT: high-resolution computed tomography; ICH: International Council for Harmonisation of Technical Requirements for Pharmaceuticals for Human Use; IPF: idiopathic pulmonary fibrosis; JRS: Japanese Respiratory Society; UIP: usual interstitial pneumonia; WOCBP: women of childbearing potential. ^#^: Stable therapy is defined as the individually and generally tolerated regimen of either nintedanib or pirfenidone (no dose changes) for at least 12 weeks prior to screening. ^¶^: WOCBP (*i.e.* fertile, following menarche and until becoming postmenopausal, unless permanently sterile). Permanent sterilisation methods include hysterectomy, bilateral salpingectomy and bilateral oophorectomy. Tubal ligation is not considered as a method of permanent sterilisation. A postmenopausal state is defined as no menses for 12 months without an alternative medical cause.

Patients with relevant airway obstruction (pre-bronchodilator forced expiratory volume in 1 s/FVC<0.7) at screening, acute exacerbation of IPF within 12 weeks prior to screening and/or during the screening period (investigator-determined), being treated with immunosuppressive medications (other than oral corticosteroids) or prednisone >15 mg·day^−1^ or equivalent for respiratory or pulmonary reasons, or having a lower respiratory tract infection requiring treatment within 4 weeks prior to screening will be excluded from the trial. Patients taking a concomitant medication listed in table 2 will not be eligible for the trial. A historical chest HRCT scan will be used to determine eligibility (confirmed locally), as long as it is performed within 12 months prior to screening. If the historical scan is unavailable, or the available scan does not meet the required image acquisition specification, the HRCT scan may be performed during screening, provided all other eligibility criteria are fulfilled. If previously obtained, surgical lung biopsy or transbronchial lung cryobiopsy may be used to locally confirm IPF diagnosis.

**TABLE 2:** Concomitant medication restrictions

Medication	Prior to trial^#^	Screening period	Treatment period	Follow-up period
Strong inhibitors and inducers of CYP3A4	Permitted^¶^	Permitted^¶^	Not permitted	Not permitted
Strong inhibitors of UGTIA4	Permitted^¶^	Permitted^¶^	Not permitted	Not permitted
p-gp inhibitors and inducers	Permitted^¶^	Permitted^¶^	Not permitted	Permitted
Stem cell therapy for the treatment of pulmonary fibrosis	Not permitted	Not permitted	Not permitted	Not permitted
Immunosuppressive medications used for respiratory or pulmonary reasons (other than oral steroids)	Permitted^+^	Not permitted	Not permitted	Permitted
Prednisone >15 mg·day^−1^ or equivalent used for respiratory or pulmonary reasons^§^	Permitted	Not permitted	Not permitted^f^	Permitted

IPF: idiopathic pulmonary fibrosis; p-gp: p-glycoprotein. ^#^: Duration of restriction before screening (see exclusion criteria). ^¶^: Strong CYP3A4, p-gp inhibitors and inducers, and UGT1A4 inhibitors should not be taken within five half-life times prior to randomisation. ^+^: Referring to historical episodes of intake, permitted unless the treatment is ongoing at time of screening. ^§^: Inhaled, intranasal, intra-articular and topical corticosteroids are allowed. ^f^: Prednisone >15 mg·day^−1^ or equivalent can be prescribed during the treatment period in case of (suspected) acute IPF exacerbation and other underlying conditions.

### End-points

The primary end-point is the annual rate of decline in FVC (mL·year^−1^) assessed over a treatment period up to a maximum of 52 weeks. The secondary end-point is the absolute change from baseline in FVC (mL) at week 24. Further end-points include time to clinically meaningful events (disease progression or initiation of background treatment), patient-reported outcomes, additional lung function end-points for FVC and *D*_L__CO_, exploratory HRCT assessment (absolute change from baseline in quantitative lung fibrosis and quantitative ILD scores at week 24), and pharmacokinetic and pharmacodynamic end-points. The detailed list of end-points is reported in table 3.

**TABLE 3:** End-points

**Primary end-point**	The annual rate of decline in FVC (mL·year^−1^) assessed over a treatment period up to a maximum of 52 weeks.
**Secondary end-point**	The absolute change from baseline in FVC (mL) at week 24.
**Further end-points**	**Clinical meaningful events** Time to the first occurrence of any of the components of the composite end-point: first acute IPF exacerbation, first hospitalisation for respiratory cause, or death (whichever occurs first) over the duration of the trial. Time to initiation of background therapy over the duration of the trial (in the group of trial patients without background treatment). **Patient-reported outcomes** Absolute change from baseline in L-PF symptoms dyspnoea, cough and fatigue domain scores, symptoms total and impact score at weeks 24 and 52. Absolute change from baseline in PGI-S of shortness of breath, cough and fatigue, overall symptoms, impact of symptoms on daily life and activities, and overall health status scale scores at weeks 24 and 52. PGI-C of shortness of breath, cough, fatigue, overall symptoms, impact of symptoms on daily life and activities, and overall health status assessment status at weeks 24 and 52. **Lung function** Time to absolute decline in FVC % predicted of >10% from baseline or death up to week 52. Time to a relative decline from baseline in FVC % predicted of >10% or death up to week 52. Time to absolute decline from baseline in FVC % predicted of >5% or death up to week 52. Time to a relative decline from baseline in FVC % predicted of >5% or death up to week 52. Time to absolute decline in *D*_L__CO_ % predicted of >15% from baseline or death up to week 52. Absolute change from baseline in FVC % predicted at weeks 24 and 52. Absolute change from baseline in FVC (mL) at week 52. Absolute change from baseline in *D*_L__CO_ % predicted at weeks 24 and 52. **HRCT exploratory assessment^#^** Absolute change from baseline in QLF (%) at week 24. Absolute change from baseline in QLF (mL) at week 24. Absolute change from baseline in QILD (%) at week 24. Absolute change from baseline in QILD (mL) at week 24. **Pharmacokinetics** Descriptive statistics will be used to evaluate pre- and post-dose BI 1819479 concentrations. **Pharmacodynamics** LPA (18:2) concentration in plasma. Soluble protein biomarkers: MMP-7, KL-6, ICAM-1, CCL-18, SP-D, CA-125, CA19.9, CTGF, autotaxin. Neoepitope biomarkers: C3 M, C6 M, PRO-C3, PRO-C6.

CA-125: cancer antigen 125; CCL-18: chemokine (C-C motif) ligand 18; *D*_LCO_: diffusing capacity of the lungs for carbon monoxide; CTGF: connective tissue growth factor; FVC: forced vital capacity; HRCT: high-resolution computed tomography; ICAM-1: intercellular adhesion molecule 1; IPF: idiopathic pulmonary fibrosis; KL-6: Krebs von den Lungen 6; LPA: lysophosphatidic acid; L-PF: living with pulmonary fibrosis; MMP-7: matrix metalloproteinase 7; PGI-C: Patient's Global Impression of Change; PGI-S: Patient's Global Impression of Severity; PRO-C3: propeptide of type III collagen; PRO-C6: propeptide of type VI collagen; QILD: quantitative interstitial lung disease; QLF: quantitative lung fibrosis; SP-D: surfactant protein D. ^#^: Participation in the HRCT exploratory assessment is optional, at the discretion of the investigator and the trial patient and is not a prerequisite to participate in the trial.

### Safety

Physical examinations, vital signs, ECGs and routine safety laboratory tests will be taken at the beginning of the trial and at predetermined time points. Adverse events will be coded using the Medical Dictionary for Drug Regulatory Activities. Potential severe drug-induced liver injury will be considered as an adverse event of special interest and will be assessed as part of routine testing, but is considered a general safety topic and not related to the use of BI 1819479 or the drug class. An independent data monitoring committee (DMC) unblinded to treatment allocation will evaluate safety data on a regular basis.

### Statistical methods

This trial is exploratory and not designed for confirmatory hypothesis testing. The primary end-point, the annual rate of decline in FVC over a treatment period up to 52 weeks, will be evaluated separately for participants with and without approved background treatment (nintedanib/pirfenidone) using Bayesian multiple comparison procedures and modelling (MCPMod). The results of the two strata will be combined to reach an overall conclusion for proof of concept. Bayesian MCPMod will be performed based on random slope and intercept model estimates that account for the repeated nature of the data and covariates that include fixed, categorical effects for treatment-by-baseline intake of background treatment interaction and fixed, continuous effects of baseline FVC as well as baseline FVC-by-time and treatment-by-intake of background treatment-by-time interactions. Random effects will be included for trial patient response for both time and intercept. Covariate-adjusted fixed effect estimates of average slope for each dose group within each stratum and the se will be extracted from the fitted model and forwarded to the Bayesian MCPMod analysis.

Historical data from clinical trials in IPF and progressive pulmonary fibrosis will be incorporated for the placebo arm *via* a robust meta-analytic-predictive prior, with priors updated for final analysis to include the most up-to-date data available during trial conduct (supplementary tables S1 and S2). Vaguely informative priors will be generated for active BI 1819479 arms (with and without background treatment). Proof of concept will be based on detecting a non-flat dose–response and a minimum relevant effect of BI 1819479 *versus* placebo (Δ boundary), using a Bayesian MCPMod approach. The Bayesian p-values of the two trial patient strata will be combined using the inverse normal p-value combination function to conclude a non-flat dose–response. The maximum placebo-corrected BI 1819479 effects, averaged over significant dose–response shapes, will be compared with stratum-specific boundaries to establish a minimal relevant effect. The statistical model incorporates any available data up to 52 weeks, without requiring data from all patients at this time point.

The secondary end-point is the assessment of change in FVC between treatment groups at week 24 using a restricted maximum likelihood (REML)-based mixed model for repeated measures (MMRM). All further end-points will be considered exploratory in nature. Continuous end-points will be analysed descriptively as well as using a REML-based repeated measures approach. Time-to-event outcomes will be analysed using Cox proportional hazards models with Kaplan–Meier plots. Plasma concentrations and exploratory biomarkers of BI 1819479 will be analysed descriptively. Missing data will not be imputed in the primary end-point analysis, except for death, for which a poor outcome will be assigned. Additional details on statistical methods are available in the supplementary material.

### Sample size determination

Simulations were performed to assess the success probabilities under various scenarios. Assuming a maximum effect on FVC decline of 70 mL·year^−1^ for participants on background antifibrotic treatment and 110 mL·year^−1^ for those not on background antifibrotic treatment, a sample size of 300 is required for a probability of success (non-flat dose–response curve) of at least 83% if the proportion on background antifibrotic treatment is 67%, or 81% if the proportion on background antifibrotic treatment is 80%.

### Trial oversight and ethics approval

The DMC will recommend continuation, modification or termination of the trial, and all DMC recommendations will be reported to the appropriate regulatory authorities/health authorities, institutional review boards/independent ethics committees and to investigators as required by local law. The trial will be carried out in compliance with the protocol, the ethical principles laid down in the Declaration of Helsinki, in accordance with the International Council for Harmonisation of Technical Requirements for Pharmaceuticals for Human Use (ICH) guidelines for Good Clinical Practice (GCP), relevant Boehringer Ingelheim standard operating procedures, EU regulation 536/2014, the Japanese GCP regulations (Ministry of Health and Welfare ordinance no. 28, 27 March 1997) and other relevant regulations. Prior to participation in the trial, written informed consent will be obtained from each patient (or the patient's legally accepted representative) according to ICH GCP and the regulatory and legal requirements of the participating country.

## Discussion

### Rationale for trial

The success of LPA and autotaxin inhibitors for the treatment of IPF in clinical trials has been mixed. The phase III ISABELA-1 [23] and ISABELA-2 [24] trials with ziritaxestat, and, more recently, the phase II trial with BBT-877 [29], both small-molecule autotaxin inhibitors, were terminated early after it had been concluded that the benefit-to-risk profile of ziritaxestat no longer supported continuation and BTT-877 failed to meet its primary end-point [29]. However, cudetaxestat, a non-competitive autotaxin inhibitor, is currently in a phase II trial [20] investigating efficacy and safety with or without background treatment (nintedanib/pirfenidone) in IPF. BMS-986020, a first-generation LPA antagonist, was discontinued after a phase II trial because of liver-related adverse events, despite the drug significantly slowing the rate of FVC decline [30–32]. Following the discontinuation of BMS-986020, BMS-986278 was developed as a second-generation LPA antagonist and is currently being investigated in a phase III trial in IPF [21, 22].

LPA pathway inhibition is possible through different mechanisms and may be complementary to and/or synergistic with currently approved drugs for IPF. LPA is produced *via* the autotaxin pathway, where autotaxin converts lysophosphatidylcholine into LPA [10]. Therefore, LPA synthesis can be targeted with competitive and non-competitive autotaxin inhibitors. The LPA pathway can also be inhibited by blocking the LPA receptors, which are involved in various cellular processes [12–14].

While there are currently no approved LPA pathway inhibitors on the market for IPF, BI 1819479, which is investigated in this study, was granted an Orphan Drug Designation by the US Food and Drug Administration for the potential treatment of IPF based on preclinical data [33, 34]. Therefore, BI 1819479 may provide an additional treatment option to patients with IPF.

The inclusion of patients with and without background treatment with nintedanib/pirfenidone will provide efficacy and safety data for BI 1819479 as a monotherapy or as an add-on with available standard-of-care treatment. During the recruitment period, the number of trial patients randomised will be controlled for so that at least 20% of patients are not receiving currently approved IPF treatment, ensuring that enough patients without background treatment at baseline are included in the trial.

Treatment with BI 1819479 has the potential to provide significant benefit to patients with IPF by ultimately slowing lung function decline, improving symptoms and quality of life over a long-term period, and therefore, warrants further investigation.

### Rationale for end-points

The primary objective is to demonstrate proof of concept with respect to a non-flat dose–response curve and a minimum relevant treatment effect, as well as define a suitable dose range for BI 1819479 regarding efficacy and safety for further pivotal testing in phase III trials. A change in FVC is often considered a significant marker of disease progression and is associated with increased mortality in patients with IPF [35], and is therefore commonly used as a primary end-point in clinical trials in IPF [36–39]. Patients will be treated until either they complete 52 weeks of treatment or until 24 weeks after randomisation of the last patient to collect extended efficacy and safety data on the effect of BI 1819479 in a controlled manner. While the primary end-point is 52 weeks, the statistical model can use any available data up to 52 weeks by assuming the change from baseline in FVC to be linear over time. Data up to 52 weeks is not required for all patients. Using the annualised end-point instead of a 24-week time point reduces the required sample size by approximately 33%. This is because of the remarkably lower variability of the slope estimate from a random slope and intercept model based on data with up to 52 weeks of follow-up compared with the least squares means estimate from an MMRM based on data up to week 24 only.

Patient-reported outcomes will be assessed at visits 2 and 8 and at the end-of-treatment visit; only patients with data for the time point in question will be included in each assessment.

### Rationale for dose selection

BI 1819479 dose selection in this trial was based on data obtained from four phase I clinical trials that investigated safety, tolerability, pharmacokinetics and pharmacodynamics following single doses and multiple rising doses [25–28]. The dosing regimens for the phase II trial were selected based on several key considerations: 1) to evaluate at least one dose where the plasma LPA response is expected to be below the maximum achievable level; 2) to evaluate a maximal range of exposure to account for scenarios where the plasma LPA response may not correlate, or only partially correlate, with changes in clinical end-points; and 3) to evaluate the highest multiple-dose regimen assessed in phase I to ensure that the exposure–response plateau was captured.

### Patient input

In addition to considering previous feedback from patients and patient organisations while designing the trial, two patient steering committee meetings were conducted. During these meetings, input was received regarding trial design, assessments and feasibility.

### Limitations

The potential limitations of this trial include its small sample size, which is a common constraint in phase II trials, and the shorter treatment duration for some patients. However, this study has been able to reduce the sample size while maintaining statistical power due to use of Bayesian methodology. It is expected that the patient population and study duration will be expanded for phase III trials. Since BI 1819479 has not been tested in a broad population of patients, it is unknown if LPA inhibition may result in unknown receptor interactions. Exercise will not be evaluated as an end-point in this phase II trial, as while end-points such as the 6-min walk test can provide critical information on exercise capacity and is a strong predictor of mortality, these end-points are affected by numerous factors (including age, body size, comorbidities and the use of supplemental oxygen during the test), and the use of different methodologies across studies hinders the comparison of results [40].

### Conclusion

This trial investigates the efficacy, safety and optimal dose range of BI 1819479 in patients with IPF, both alone and in combination with currently approved IPF treatment. The results of this trial will also define suitable dosing for future phase III trials.

## Data Availability

To ensure independent interpretation of clinical trial results, and enable authors to fulfil their role and obligations under the International Committee of Medical Journal Editors criteria, Boehringer Ingelheim grants all external authors access to relevant clinical trial data. In adherence with the Boehringer Ingelheim Policy on Transparency and Publication of Clinical Trial Data, scientific and medical researchers can request access to clinical trial data, typically 1 year after the approval has been granted by major regulatory authorities or after termination of the development programme. Researchers should use the link https://vivli.org/ to request access to trial data and visit https://www.clinicalstudies.boehringer-ingelheim.com/msw/datatransparency for further information.

## Supplementary material

**Please note:** supplementary material is not edited by the Editorial Office, and is uploaded as it has been supplied by the author. Supplementary material 00973-2025.SUPPLEMENT Infographic 00973-2025.SUPPLEMENT2

## References

[1] Raghu G, Remy-Jardin M, Myers JL, et al. Diagnosis of idiopathic pulmonary fibrosis. An official ATS/ERS/JRS/ALAT clinical practice guideline. Am J Respir Crit Care Med 2018; 198: e44–e68. doi:10.1164/rccm.201807-1255ST 30168753

[2] Ley B, Collard HR, King TE, Jr. Clinical course and prediction of survival in idiopathic pulmonary fibrosis. Am J Respir Crit Care Med 2011; 183: 431–440. doi:10.1164/rccm.201006-0894CI 20935110

[3] US Food and Drug Administration. OFEV® (nintedanib): prescribing information. 2022. Date last accessed: 30 June 2025. Date last updated: January 2022. www.accessdata.fda.gov/drugsatfda_docs/label/2022/205832Orig1s016lbl.pdf

[4] Raghu G, Remy-Jardin M, Richeldi L, et al. Idiopathic pulmonary fibrosis (an update) and progressive pulmonary fibrosis in adults: an official ATS/ERS/JRS/ALAT Clinical Practice Guideline. Am J Respir Crit Care Med 2022; 205: e18–e47. doi:10.1164/rccm.202202-0399ST 35486072 PMC9851481

[5] US Food and Drug Administration. ESBRIET® (pirfenidone): prescribing information. 2023. Date last accessed: 12 December 2024. Date last updated: February 2023. www.accessdata.fda.gov/drugsatfda_docs/label/2019/022535s012,208780s002lbl.pdf

[6] Maher TM, Strek ME. Antifibrotic therapy for idiopathic pulmonary fibrosis: time to treat. Respir Res 2019; 20: 205. doi:10.1186/s12931-019-1161-4 31492155 PMC6731623

[7] Culver DA, Behr J, Belperio JA, et al. Patient registries in idiopathic pulmonary fibrosis. Am J Respir Crit Care Med 2019; 200: 160–167. doi:10.1164/rccm.201902-0431CI 31034241 PMC6635784

[8] Strongman H, Kausar I, Maher TM. Incidence, prevalence, and survival of patients with idiopathic pulmonary fibrosis in the UK. Adv Ther 2018; 35: 724–736. doi:10.1007/s12325-018-0693-1 29644539 PMC5960490

[9] Fernandez Perez ER, Daniels CE, Schroeder DR, et al. Incidence, prevalence, and clinical course of idiopathic pulmonary fibrosis: a population-based study. Chest 2010; 137: 129–137. doi:10.1378/chest.09-1002 19749005 PMC2803118

[10] Magkrioti C, Galaris A, Kanellopoulou P, et al. Autotaxin and chronic inflammatory diseases. J Autoimmun 2019; 104: 102327. doi:10.1016/j.jaut.2019.102327 31471142

[11] Zulfikar S, Mulholland S, Adamali H, et al. Inhibitors of the autotaxin-lysophosphatidic acid axis and their potential in the treatment of interstitial lung disease: current perspectives. Clin Pharmacol 2020; 12: 97–108. doi:10.2147/CPAA.S228362 32765123 PMC7367740

[12] Volkmann ER, Denton CP, Kolb M, et al. Lysophosphatidic acid receptor 1 inhibition: a potential treatment target for pulmonary fibrosis. Eur Respir Rev 2024; 33: 240015. doi:10.1183/16000617.0015-2024 39009409 PMC11262619

[13] Tager AM, LaCamera P, Shea BS, et al. The lysophosphatidic acid receptor LPA1 links pulmonary fibrosis to lung injury by mediating fibroblast recruitment and vascular leak. Nat Med 2008; 14: 45–54. doi:10.1038/nm1685 18066075

[14] Salgado-Polo F, Perrakis A. The structural binding mode of the four autotaxin inhibitor types that differentially affect catalytic and non-catalytic functions. Cancers 2019; 11: 1577. doi:10.3390/cancers11101577 31623219 PMC6826961

[15] Nathan S, Zhang H, Andreoli M, et al. CREB-dependent LPA-induced signaling initiates a pro-fibrotic feedback loop between small airway basal cells and fibroblasts. Respir Res 2021; 22: 97. doi:10.1186/s12931-021-01677-0 33794877 PMC8015171

[16] Stortelers C, Kerkhoven R, Moolenaar WH. Multiple actions of lysophosphatidic acid on fibroblasts revealed by transcriptional profiling. BMC Genomics 2008; 9: 387. doi:10.1186/1471-2164-9-387 18702810 PMC2536681

[17] Jiang S, Yang H, Li M. Emerging roles of lysophosphatidic acid in macrophages and inflammatory diseases. Int J Mol Sci 2023; 24: 12524. doi:10.3390/ijms241512524 37569902 PMC10419859

[18] Maher TM, Ford P, Brown KK, et al. Ziritaxestat, a novel autotaxin inhibitor, and lung function in idiopathic pulmonary fibrosis: the ISABELA 1 and 2 randomised clinical trials. JAMA 2023; 329: 1567–1578. doi:10.1001/jama.2023.5355 37159034 PMC10170340

[19] Maher T, Song JW, Kramer MR, et al. Phase 2 study design and analysis approach for BBT-877: an autotaxin inhibitor targeting idiopathic pulmonary fibrosis. BMJ Open Respir Res 2025; 12: e003038. doi:10.1136/bmjresp-2024-003038 PMC12097056 40404183

[20] ClinicalTrials.gov. RESPIRARE - efficacy and safety of cudetaxestat in patients with idiopathic pulmonary fibrosis (IPF). NCT05373914. Date last accessed: 12 December 2024. Date last updated: 18 May 2022. https://clinicaltrials.gov/study/NCT05373914

[21] ClinicalTrials.gov. A study to evaluate the efficacy, safety, and tolerability of bms-986278 in participants with idiopathic pulmonary fibrosis. NCT06003426. Date last accessed: 12 December 2024. Date last updated: 22 August 2024. https://clinicaltrials.gov/study/NCT06003426

[22] Corte TJ, Cottin V, Glassberg MK, et al. BMS-986278, an oral lysophosphatidic acid receptor 1 (LPA1) antagonist, for patients with idiopathic pulmonary fibrosis: results from a phase 2 randomised trial. Am J Respir Crit Care Med 2023; 211: A2785. doi:10.1164/rccm.202405-0977OC

[23] ClinicalTrials.gov. A clinical study to test how effective and safe GLPG1690 is for subjects with idiopathic pulmonary fibrosis (IPF) when used together with standard of care (ISABELA1). NCT03711162. Date last accessed: 19 May 2025. Date last updated: 29 July 2022. https://clinicaltrials.gov/study/NCT03711162

[24] ClinicalTrials.gov. A clinical study to test how effective and safe GLPG1690 is for participants with idiopathic pulmonary fibrosis (IPF) when used together with standard of care (ISABELA2). NCT03733444. Date last accessed: 19 May 2025. Date last updated: 29 July 2022. https://clinicaltrials.gov/study/NCT03733444

[25] ClinicalTrials.gov. A study to test how healthy women tolerate different doses of BI 1819479. NCT05440799. Date last accessed: 12 December 2024. Date last updated: 14 August 2023. https://clinicaltrials.gov/study/NCT05440799

[26] ClinicalTrials.gov. A study in healthy men to test how esomeprazole influences the amount of BI 1819479 in the blood. NCT05467475. Date last accessed: 12 December 2024. Date last updated: 26 January 2023. https://clinicaltrials.gov/study/NCT05467475

[27] ClinicalTrials.gov. A study in healthy men to test how well different doses of BI 1819479 are tolerated and taken up by the body with or without food. NCT04801693. Date last accessed: 12 December 2024. Date last updated: 4 May 2022. https://clinicaltrials.gov/study/NCT04801693

[28] ClinicalTrials.gov. A study in healthy men to test how well different doses of BI 1819479 are tolerated. NCT05469646. Date last accessed: 12 December 2024. Date last updated: 5 April 2023. https://clinicaltrials.gov/study/NCT05469646

[29] ClinicalTrials.gov. To evaluate the efficacy, safety, and tolerability of BBT-877 in patients with IPF. NCT05483907. Date last accessed: 12 December 2024. Date last updated: 13 August 2024. https://clinicaltrials.gov/study/NCT05483907

[30] ClinicalTrials.gov. Safety and efficacy of a lysophosphatidic acid receptor antagonist in idiopathic pulmonary fibrosis. NCT01766817. Date last accessed: 12 December 2024. Date last updated: 11 August 2020. https://clinicaltrials.gov/study/NCT01766817

[31] Palmer SM, Snyder L, Todd JL, et al. Randomised, double-blind, placebo-controlled, phase 2 trial of BMS-986020, a lysophosphatidic acid receptor antagonist for the treatment of idiopathic pulmonary fibrosis. Chest 2018; 154: 1061–1069. doi:10.1016/j.chest.2018.08.1058 30201408

[32] Decato BE, Leeming DJ, Sand JMB, et al. LPA(1) antagonist BMS-986020 changes collagen dynamics and exerts antifibrotic effects in vitro and in patients with idiopathic pulmonary fibrosis. Respir Res 2022; 23: 61. doi:10.1186/s12931-022-01980-4 35303880 PMC8933988

[33] Boehringer Ingelheim. US FDA Grants BI 1819479 orphan drug designation for idiopathic pulmonary fibrosis. 2023. Date last accessed: 29 August 2024. www.boehringer-ingelheim.com/us/investigational-compound-granted-fda-orphan-drug-designation

[34] Jia Y, Li Y, Xu XD, et al. Design and development of autotaxin inhibitors. Pharmaceuticals 2021; 14: 1203. doi:10.3390/ph14111203 34832985 PMC8622848

[35] Richeldi L, Azuma A, Cottin V, et al. Design of a phase III, double-blind, randomised, placebo-controlled trial of BI 1015550 in patients with idiopathic pulmonary fibrosis (FIBRONEER-IPF). BMJ Open Respir Res 2023; 10: e001563. doi:10.1136/bmjresp-2022-001563 PMC10441083 37597969

[36] Paterniti MO, Bi Y, Rekic D, et al. Acute exacerbation and decline in forced vital capacity are associated with increased mortality in idiopathic pulmonary fibrosis. Ann Am Thorac Soc 2017; 14: 1395–1402. doi:10.1513/AnnalsATS.201606-458OC 28388260

[37] Richeldi L, Azuma A, Cottin V, et al. Trial of a preferential phosphodiesterase 4B inhibitor for idiopathic pulmonary fibrosis. N Engl J Med 2022; 386: 2178–2187. doi:10.1056/NEJMoa2201737 35569036

[38] King TE Jr., Bradford WZ, Castro-Bernardini S, et al. A phase 3 trial of pirfenidone in patients with idiopathic pulmonary fibrosis. N Engl J Med 2014; 370: 2083–2092. doi:10.1056/NEJMoa1402582 24836312

[39] Richeldi L, du Bois RM, Raghu G, et al. Investigators IT. Efficacy and safety of nintedanib in idiopathic pulmonary fibrosis. N Engl J Med 2014; 370: 2071–2082. doi:10.1056/NEJMoa1402584 24836310

[40] Lancaster LH. Utility of the six-minute walk test in patients with idiopathic pulmonary fibrosis. Multidiscip Respir Med 2018; 13: 45. doi:10.1186/s40248-018-0158-z 30559965 PMC6291931

