# Supplementary material for: Design of a phase II randomised, double-blind, placebo-controlled, dose-finding trial of BI 1819479 in patients with idiopathic pulmonary fibrosis
Source: ERJ Open Res. 2026 Mar 16;12(2):00973-2025. doi: 10.1183/23120541.00973-2025 (PMC12991008; doi:10.1183/23120541.00973-2025)
Supplement: Supplementary file 1 [file 00973-2025.SUPPLEMENT.pdf]

Supplementary Materials

Design of a Phase II randomised, double-blind, placebo-controlled, dose-finding trial of BI 1819479 in patients with idiopathic pulmonary fibrosis

Author list: Wim A Wuyts,[1,2] Francesco Bonella,[3] Haruyuki Ishii,[4] Joyce S Lee,[5] Elisabetta Renzoni,[6,7] Sandra Hadl,[8] Julia Krzykalla,[9] Susanne Stowasser,[10] Michael Engel,[10] Maria Molina Molina[11]

Affiliations: [1]Unit for Interstitial Lung Diseases, Department of Respiratory Diseases, University Hospitals Leuven, Leuven, Belgium; [2]Department of Chronic Diseases, Metabolism, and Ageing, KU Leuven, Leuven, Belgium; [3]Center for Interstitial and Rare Lung Diseases, Pneumology Department, Ruhrlandklinik, University Hospital, University of Essen, European Reference Network (ERN)-LUNG, ILD Core Network, Essen, Germany; [4]Department of Respiratory Medicine, Kyorin University Faculty of Medicine, Mitaka City, Japan; [5]University of Colorado, School of Medicine, Department of Medicine, Aurora, CO, United States; [6]Interstitial Lung Disease Unit, Royal Brompton and Harefield Clinical Group, Guy's and St Thomas' NHS Foundation Trust, London, UK; [7]Margaret Turner Warwick Centre for Fibrosing Lung Disease, National Heart and Lung Institute, Imperial College London, London, UK; [8]Boehringer Ingelheim RCV GmbH & Co KG, Vienna, Austria; [9]Boehringer Ingelheim Pharma GmbH & Co. KG, Biberach an der Riss, Germany; [10]Boehringer Ingelheim Corporation, Ingelheim am Rhein, Germany; [11]Interstitial Lung Disease Unit, Respiratory Department, University Hospital of Bellvitge, IDIBELL, Hospitalet de Llobregat (Barcelona), CIBERES, Barcelona, Spain.

Supplementary Methods

Additional information on treatment discontinuation or modification {11b}

Patients are encouraged to attend all remaining scheduled trial visits after permanent discontinuation of trial treatment—even in the absence of further treatment administration—to support ongoing safety monitoring and data collection. If patients are unwilling to attend scheduled visits, investigators will discuss alternative options, such as contacting patients at scheduled visit time points to collect information on adverse events, concomitant medications, and vital status. Completion of both the end-of-treatment and end-of-trial visits are required for all patients. Any clinically relevant abnormal values (investigator-assessed) identified at the final visit will be monitored using appropriate follow-up testing until they return to medically acceptable levels. Adverse events will be followed up until resolution, assessed as “chronic” or “stable”, or until no further information can be obtained.

Additional information on treatment adherence {20c}

Trial participants are requested to bring all remaining trial investigational medicinal product, including empty package material and in-use wallets, with them when attending visits.

Based on tablet counts, treatment compliance will be calculated as shown in the formula below:

$$Treatment\ compliance\ (\%) = \frac{\text{Number of tablets actually taken x 100}}{\begin{array}{c} Number\ of\ tablets\ which\ should\ have\ been \\ taken\ as\ directed\ by\ investigator \end{array}}$$

If the number of doses taken is not between 80-120%, site staff will explain to the trial participant the importance of treatment compliance. Additionally, participants are

required to fill out a medication diary to document their adherence to the treatment regimen 7 days prior to the next visit. The diary will include sections for documenting the date and time of trial medication intake, modifications, as well as general well-being and notes on missed doses.

Additional data on the statistical methods {20a}

Bayesian MCPMod allows for the consideration of multiple potential dose–response patterns. The following models have been selected to cover a plausible yet diverse range of potential dose–response patterns: linear, Emax, exponential and sigmoid Emax. Historical data from prior clinical trials conducted in idiopathic pulmonary fibrosis (IPF) and progressive pulmonary fibrosis (PPF) (Tables S1 and S2) will inform the placebo arm using a robust meta-analytic predictive prior. For final analysis, priors will be updated to incorporate the most up-to-date data as more will become available during trial conduct, e.g. FIBRONEER-IPF [NCT05321069], STARSCAPE [NCT04552899], ZEPHYRUS I and II [NCT03955146 and NCT04419558, respectively].

To evaluate a non-flat dose–response curve in each stratum, a Bayesian version of the MCPMod testing procedure will be used. This test accounts for multiplicity due to the set of candidate dose–response shapes and identifies the significant candidate models amongst them.

The mixed model for repeated measures (MMRM) used for the secondary endpoint at Week 24 will be fitted using all data available. The model will include fixed, categorical effects of treatment at each visit, baseline intake of background treatment at each visit, and fixed, continuous effects of baseline FVC value at each visit. Visits will be treated as the repeated measure, with an unstructured covariance structure

used to model within-trial patient measurements. Treatment comparisons will be the contrasts between placebo and the different BI 1819479 doses at each measured time point. Any p-values presented for the secondary endpoint will be considered nominal in nature, with no adjustment for multiplicity.

Further endpoints will be considered exploratory. Continuous endpoints will also be analysed using a REML-based repeated measures approach, with fixed continuous effects for the baseline value of the respective endpoint at each visit instead of baseline FVC value.

Time-to-event endpoints will be analysed with a Cox proportional hazards model using data over the whole trial. The model will include treatment and baseline intake of background treatment as covariates. Kaplan–Meier plots by treatment group will be presented.

Statistical methods to handle missing data {20c}

Missing data will not be imputed in the primary endpoint analysis except for death, for which a poor outcome will be assigned. The random slope and intercept model will handle missing data based on a likelihood method under the “missing at random assumption” and linearity-over-time assumption. The mixed-effect model will handle missing data based on a likelihood method under the "missing at random assumption". In the analysis of all other continuous endpoints, missing data will not be imputed. In the analysis of time-to-event endpoints, missing or incomplete data will be handled using standard survival analysis techniques (i.e. censoring).

Additional information on biological samples {26b, 33}

Sample and data usage must be in accordance with the separate biobanking informed consent. The Boehringer Ingelheim internal facilities that will be storing the

biological samples from clinical trial participants, as well as the external banking facility, are qualified for the storage of biological samples collected in clinical trials. An appropriate sample and data management system, including audit trail for clinical data and samples to identify and destroy such samples according to the informed consent form, is in place. A ‘fit for the purpose’ documentation (biomarker proposal, analysis plan and report) ensures compliant usage. A ‘fit for the purpose’ approach will be used for assay/equipment validation depending on the intended use of the biomarker data.

Figure S1. Geographic distribution of participating countries in the trial

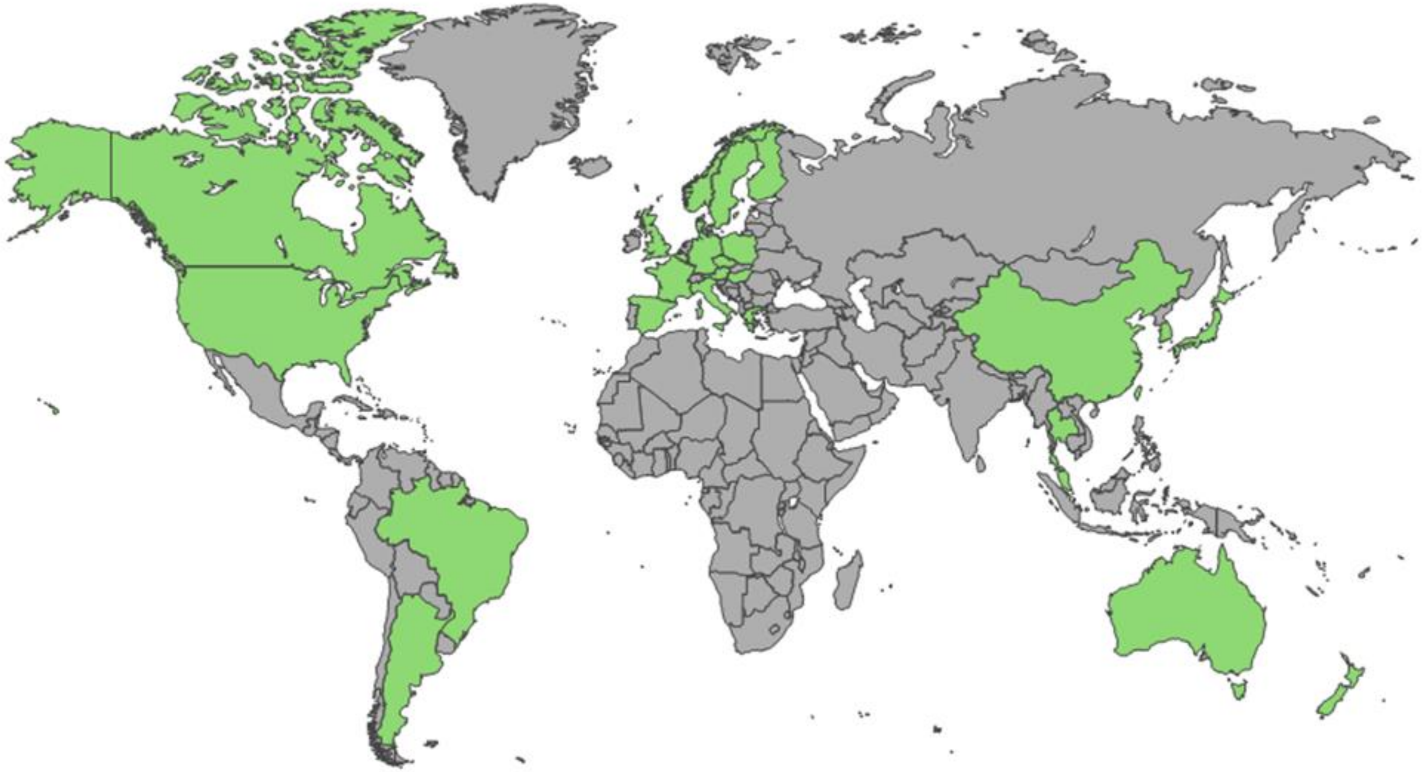

This study will take place in Argentina, Australia, Austria, Belgium, Brazil, Canada, China, Czech Republic, Denmark, Finland, France, Germany, Greece, Hungary, Italy, Japan, Korea, Malaysia, New Zealand, Norway, Poland, Singapore, Spain, Sweden, Taiwan, Thailand, United Kingdom and United States.

Table S1. Estimated adjusted means and SE for the placebo group of trial patients without background treatment

Trial	N	Adjusted rate of decline in FVC over 52 weeks, mL/year (SE)
INPULSIS-1 (NCT01335464)	204	−240.22 (19.065)
INPULSIS-2 (NCT01335477)	217	−199.35 (19.422)
NCT01979952	54	−156.13 (91.618)
TOMORROW (NCT00514683)	84	−206.63 (37.195)
INBUILD (NCT02999178)[a]	206	−211.07 (20.49)
ISABELA 1 (NCT03711162) and ISABELA 2 (NCT03733444) (without background treatment)[b]	126	−149.1 (25.74)
BMS-986278 (NCT04308681) (without background treatment)[b]	30	−128.64 (89.29)

FVC, forced vital capacity; SE, standard error; UIP, usual interstitial pneumonia.

Based on unpublished data. Some trials were reanalysed using the analysis model planned for the primary analysis and some results were taken directly from the respective clinical trial reports.

For final analysis, priors will be updated to incorporate the most up-to-date data as more will become available during trial conduct, e.g. FIBRONEER-IPF [NCT05321069], STARSCAPE [NCT04552899], ZEPHYRUS I and II [NCT03955146 and NCT04419558, respectively].

[a]UIP-like fibrotic pattern only.

[b]Estimates for primary endpoint (rate of change in per cent predicted FVC from baseline to Week 26) are transformed to change from baseline in FVC (mL) using baseline values and extrapolated from 26 weeks to 52 weeks.

Table S2. Estimated adjusted means and SE for the placebo group of trial patients with background treatment

Trial	N	Adjusted rate of decline in FVC over 52 weeks, mL/year (SE)
ISABELA 1 (NCT03711162) and ISABELA 2 (NCT03733444) (background pirfenidone)	149	−189.00 (21.760)
ISABELA 1 (NCT03711162) and ISABELA 2 (NCT03733444) (background nintedanib)	147	−163.10 (21.735)
BMS-986278 (NCT04308681) (background antifibrotic treatment)[a]	62	−221.54 (48.98)

FVC, forced vital capacity; SE, standard error.

For final analysis, priors will be updated to incorporate the most up-to-date data as more will become available during trial conduct, e.g. FIBRONEER-IPF [NCT05321069], STARSCAPE [NCT04552899], ZEPHYRUS I and II [NCT03955146 and NCT04419558, respectively].

[a]Estimates for the primary endpoint (rate of change in FVC % predicted from baseline to Week 26) are transformed to change from baseline in FVC (mL) using baseline values and extrapolated from 26 weeks to 52 weeks.
