# Supplementary material for: Design of a phase II randomised, double-blind, placebo-controlled, dose-finding trial of BI 1819479 in patients with idiopathic pulmonary fibrosis
Source: ERJ Open Res. 2026 Mar 16;12(2):00973-2025. doi: 10.1183/23120541.00973-2025 (PMC12991008; doi:10.1183/23120541.00973-2025)
Supplement: Supplementary file 2 [file 00973-2025.SUPPLEMENT2.pdf]

Design of a Phase II dose-finding trial of BI 1819479 in patients with IPF

Trial medication

Why is BI 1819479 being studied?

Idiopathic pulmonary fibrosis (IPF) is a lung disease that causes scarring and inflammation in the lungs. Researchers have found that blocking a specific pathway called the lysophosphatidic acid (LPA) axis might help treat IPF. BI 1819479 blocks the LPA pathway, and has been tested in animals and healthy humans, with the results showing that it does not cause any unexpected adverse events

LPA axis

BI 1819479

Lung scarring

This trial will test how effective BI 1819479 is at treating IPF and see if it causes any side effects

Trial participants

Who will be taking part in the trial?

BI 1819479 will be tested in a Phase II trial and people included must have certain characteristics:

- ✓ **IPF** and aged **40+ years**
- ✗ Use of **immunosuppressive medication**
- ✓ Either **taking nintedanib or pirfenidone** for at least 12 weeks prior to the start of treatment and planning to continue taking them during the trial, **or not taking these approved treatments** for at least 12 weeks prior to the start of treatment*

This trial is taking place at approximately 200 sites in 28 countries around the world

Trial design

What will the trial involve?

People take tablets of dose 1, dose 2 or dose 3 of BI 1819479, or placebo

- Dose 1 of BI 1819479
- Dose 2 of BI 1819479
- Dose 3 of BI 1819479
- Placebo

At least 24 weeks

All people will take BI 1819479 or placebo for a minimum of **24 weeks** and will have **8 trial site visits (including screening)**

People will then continue taking BI 1819479 or placebo up to a maximum of **52 weeks** with **3 more trial site visits**

Trial outcomes

What will the trial be looking at?

Lung function

Whether up to 52 weeks of treatment with BI 1819479 can **slow down or prevent a decline in lung function** compared with placebo

IPF worsening

Whether BI 1819479 has an effect on the time from taking treatment to **IPF worsening or starting other approved treatments, and how patients cope with IPF**

Medical issues (adverse events)

Any **new medical issues (adverse events)** that people experience while taking BI 1819479 or placebo

IPF=idiopathic pulmonary fibrosis; LPA=lysophosphatidic acid.

* Combination of nintedanib plus pirfenidone is not allowed. Already taking treatment: taking either nintedanib or pirfenidone for at least 12 weeks before start of trial treatment and planning to stay on this treatment during the trial. Not already on treatment: not taking nintedanib or pirfenidone for at least 12 weeks prior to start of trial treatment (either never taken nintedanib or pirfenidone, or have taken previously and then stopped), and not planning to start or re-start.
